# Assessing of the use of proteins A, G, and chimeric protein AG to detect marine mammal immunoglobulins

**DOI:** 10.1371/journal.pone.0291743

**Published:** 2023-09-21

**Authors:** Michael Essien Sakyi, Takashi Kamio, Kaoru Kohyama, Md. Matiur Rahman, Kaori Shimizu, Ayaka Okada, Yasuo Inoshima

**Affiliations:** 1 Cooperative Department of Veterinary Medicine, Laboratory of Food and Environmental Hygiene, Gifu University, Gifu, Japan; 2 Joint Graduate School of Veterinary Sciences, Gifu University, Gifu, Japan; 3 Port of Nagoya Public Aquarium, Nagoya, Aichi, Japan; 4 Izu Mito Sea Paradise, Numazu, Shizuoka, Japan; 5 Faculty for Veterinary, Department of Medicine, Animal and Biomedical Sciences, Sylhet Agricultural University, Sylhet, Bangladesh; 6 Education and Research Center for Food Animal Health, Gifu University (GeFAH), Gifu, Japan; Animal Health Centre, CANADA

## Abstract

In recent years, there has been an increase in infectious diseases in marine mammals, including brucellosis, infections of morbillivirus, herpesvirus, and poxvirus. Several serological diagnostic methods, including enzyme-linked immunosorbent assays, immunofluorescence assays (ELISA), and western blotting, have been used to detect antibodies against pathogens in marine mammals. However, options for commercial secondary antibodies used to detect antibodies in marine mammals are limited; therefore, the use of proteins A, G, or chimeric protein AG may provide a suitable alternative. This study aimed to assess the use of proteins A, G, and chimeric protein AG to detect marine mammal immunoglobulins. Currently, there are no comparative studies on the use of proteins A, G, and chimeric protein AG for the detection of immunoglobulins in marine mammals. In this study, we used ten pinnipeds’ species (Baikal seal, California sea lion, harbor seal, northern fur seal, ringed seal, South American fur seal, South American sea lion, spotted seal, Steller sea lion, and walrus) and five cetacean species (beluga whale, bottlenose dolphin, harbor porpoise, killer whale, and Pacific white-sided dolphin) and compare binding ability to proteins A, G, or chimeric protein AG by ELISA. The results revealed that the immunoglobulins from pinniped and cetacean species reacted more strongly to protein A than protein G. In addition, the immunoglobulins of pinnipeds and cetaceans showed a strong binding ability to chimeric protein AG. These results suggest that proteins A, G, and chimeric protein AG would be used to help further develop serological assays.

## Introduction

Over the past two decades, several emerging infectious diseases (EIDs), including COVID-19, Ebola hemorrhagic fever, and Mpox infections, have adversely affected wildlife and the environment [1–3]. Notably, most EIDs are zoonotic; primary transmission occurs from wildlife and their prevalence has increased [4,5]. Marine mammals have shown an increase in infectious diseases such as infections of adenovirus in bottlenose dolphins (*Tursiops truncatus*) [6]; brucella species in striped dolphin (*Stenella coeruleoalba*), pygmy sperm whales (*Kogia breviceps*), killer whales (*Orincus orca*), rough-toothed dolphins (*Steno bredanensis*), and Fraser’s dolphins (*Lagenodelphis hosei*) [7]; coronavirus in beluga whales (*Delphinapterus leucas*) [8]; herpesvirus in fin whales (*Balaenoptera physalus*) and minke whales (*Balaenoptera acutorostrata*) [9]; influenza A virus in harbour seals (*Phoca vitulina*) [10]; morbillivirus in Baikal seals (*Phoca siberica*) [11], grey seals (*Halichoerus grypus*) [12,13], striped dolphins [14]; and poxvirus in harbor porpoises (*Phocoena phocoena*), short-beaked common dolphins (*Delphinus delphis*), and striped dolphins [15], and spotted seals (*Phoca largha*) [16]. Moreover, some of these viruses have been transmitted to humans and are genetically closely related to human viruses, including infections of sealpox virus [17,18] and influenza A virus [19].

In the last decade, the detection and characterization of infectious diseases in marine mammals with clinical signs have increased owing to diagnosis using polymerase chain reaction and electron microscopy [16]. A previous study reported that a marine worker who had been bitten on the hand by a captive grey seal was identified as having sealpox from the captive grey seal despite showing no clinical signs [17], suggesting that marine mammals are infected with possible zoonotic pathogens with no clinical signs. Therefore, diagnosing marine mammals with no clinical signs will help detect infectious diseases and avoid transmitting infectious diseases within marine mammals and from marine mammals to humans. However, there are currently limited or ineffective serodiagnostic tests for diagnosing marine mammals with no clinical signs.

Serodiagnostic tests help comprehend the prevalence of infectious pathogens in wildlife. Several serodiagnostic tests have been used to detect antibodies against pathogens in wildlife, including enzyme-linked immunosorbent assay (ELISA), immunofluorescence assay, western blotting (WB), agar gel immunodiffusion, virus-neutralization, and hemagglutination inhibition tests [20,21]. However, one area of improvement with many serodiagnostic tests in diverse wildlife is the relative need for reagents [22].

Previous studies reported that staphylococcal protein A and streptococcal protein G have the biological property of binding to a wide range of animals’ immunoglobulins except for birds [23–26]. Proteins A and G have different affinities in different animal species. Protein A reacts strongly with immunoglobulins in some animals, including baboons, dogs, guinea pigs, hamsters, vervet monkeys, pigs, and rabbits [24,25]. In contrast, protein G reacts strongly with cows, goats, mice, rabbits, rats, and sheep immunoglobulins [26]. The chimeric construct protein AG, which combines the immunoglobulin-binding activities of proteins A and G, has been developed [27] and has shown a strong affinity for immunoglobulins in baboons, buffaloes, cows, dogs, guinea pigs, pigs, rabbits, sheep, and vervet monkeys [24]. In wildlife, chimeric protein AG has been used in ELISA to detect antibodies against *Borrelia burgdorferi* in fallow deer and mouflons [28] and parapoxvirus in Japanese black bear, Japanese monkeys, Japanese serows, and Japanese wild boar [29]. Additionally, chimeric protein AG has been used to detect antibodies against calicivirus in Steller sea lions (*Eumetopias jubatus*) [30], anellovirus in California sea lions (*Zalophus californianus*) [31], and parapoxvirus in spotted seals [32]. Moreover, there are currently no comparative studies on the binding ability of immunoglobulins in marine mammals to proteins A, G, and chimeric protein AG.

The present study aimed to assess the binding abilities of immunoglobulins of ten pinniped and five cetacean species to proteins A, G, or chimeric protein AG. Significantly, immunoglobulin-binding proteins could be employed in marine mammals’ serological analyses of infectious diseases.

## Materials and methods

### Ethics statement

Experiments were performed in accordance with the relevant guidelines and regulations of the Gifu University Animal Care and Use Committee (approval numbers 2020–089, 2020–264, 2020–265, and 2021–227). Written informed consent was obtained from all participants.

### Collection of samples

First, to determine the dilution factor of horseradish peroxidase (HRP)-conjugated proteins A, G, and chimeric protein AG, sera from a pig, cow, and goat and plasma from a dog were collected from four individual animals with no clinical signs at Yanagido Farm and the Animal Medical Center, Gifu University (S1 and S2 Tables). Pig sera and dog plasma were used as immunoglobulins that are known to bind strongly to protein A [24]. In contrast, immunoglobulins of cows and goats are known to bind strongly to protein G [26]. Therefore, these four animals were used for determination of dilutions factors of three proteins. Next, for assessing of immunoglobulins of marine mammals, sera from ten pinniped species (Baikal seal, California sea lion, harbor seal, northern fur seal, ringed seal, South American fur seal, South American sea lion, spotted seal, Steller sea lion, and walrus) and five cetacean species (beluga whale, bottlenose dolphin, harbor porpoise, killer whale, and Pacific white-sided dolphin) were collected. The marine mammal species used were under veterinary care and sera samples were store at -80°C until used in ELISA experiment (S3 and S4 Tables).

### Determination of the dilution factor of proteins

HRP-conjugated protein A (689202; BioLegend, San Diego, CA, USA), protein G (18–161; EMD Millipore Corporation, Temecula, CA, USA), and chimeric protein AG (32490; Invitrogen, Rockford, IL, USA) were used. To select the optical density (OD) value in ELISA among HRP-conjugated proteins A, G, and chimeric protein AG, these proteins were serially diluted with phosphate buffer saline containing 0.05% Tween-20 (PBS-T) and 1% Block ACE (UK-B40, DS Pharma Biomedical, Sapporo, Japan) from 1:4000–1:128,000 dilutions. The sera/plasma of these animals were subjected to ELISA as described below and the appropriate dilution factor was determined.

### ELISA

First, to select the OD values among HRP-conjugated proteins A, G, and chimeric protein AG, animal sera or plasma from pigs, cows, goats, or dogs were diluted with 0.05 M carbonate-bicarbonate buffer (0.035 M sodium bicarbonate and 0.015 M sodium carbonate; pH 9.6) at a dilution of 1:100 [32]. Ninety-six-well microplates (U96 MAXISORP, Thermo Fisher Scientific, Roskilde, Denmark) were coated with 50 μL per well (two wells for each animal serum sample) of diluted animal sera or plasma. Plates were incubated at 37°C for 2 h. Further, plates were washed three times using 200 μL per well of PBS-T and blocked with 200 μL per well of PBS-T with 1% Block ACE by incubation at 37°C for 1 h and washed three times with PBS-T. Each well was added with 50 μL of the proteins A, G, and AG that had been serially diluted (1:4000–1:128,000 dilution) before incubating at 37°C for 1 h. After incubation, the plates were washed three times with PBS-T. Fifty microliters per well of KPL ABTS peroxidase substrate system (2, 2’-azino-di (3-ethylbenzthiazoline-6-sulfonate), 5120–0032; SeraCare Life Sciences, Milford, MA, USA) was added and incubated at 37°C for 30 min for color development. The reaction was stopped with 50 μL per well of 5% sodium dodecyl sulfate. The absorbance was measured immediately at 405 nm using a Multiskan Fc spectrophotometer (N07710; Thermo Fisher Scientific, Shanghai, China). To obtain the OD values from the two wells, the OD values of animal serum samples were subtracted from each plate’s background (blank) OD. The experiment was independently repeated three times, and the mean OD values were determined from the three replicates.

After selecting for the OD values among the three proteins, ELISA was performed to detect immunoglobulins of marine mammals, as mentioned above.

### Statistical analysis

Data for OD values in ELISA are expressed as means ± standard deviation (SD). Significant differences in the mean OD values of protein dilutions at a 95% confidence level were analyzed using the Student’s *t*-test. The corrected *p*-value cut-off was set at 0.05.

## Results

**Fig 1:**
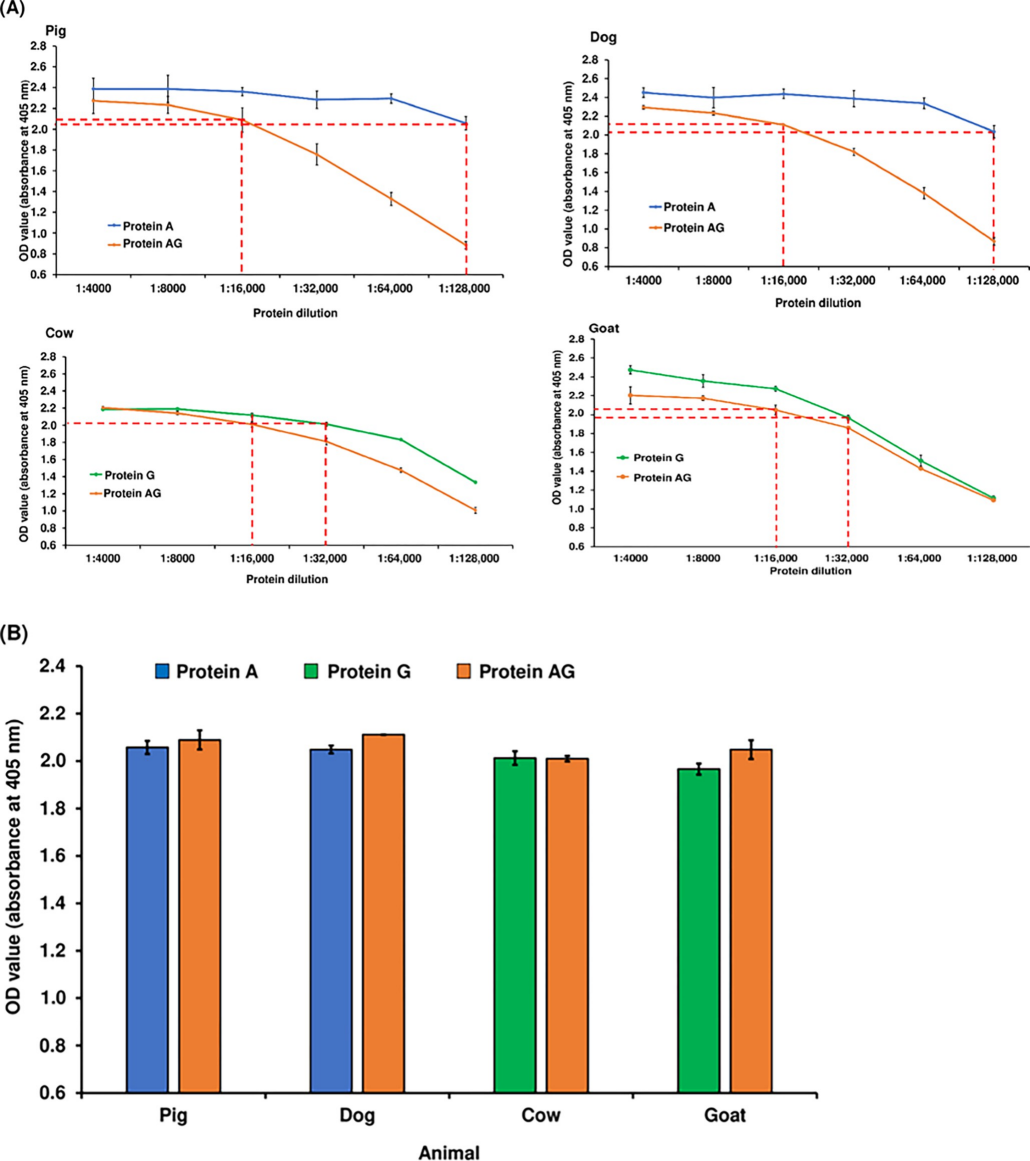
Determination of dilution factor of three proteins. (A) Mean OD values of animals’ sera/plasma with corresponding protein dilutions. The broken red lines indicate a similar mean OD value (corresponding to the dilution factor) between proteins A and AG and between proteins G and AG. Protein dilutions of 1:128,000 for protein A, 1:32,000 for protein G, and 1:16,000 for protein AG were used in ELISA for the same enzyme activity. The dilution factor of 1:128,000, 1:32,000, or 1:16,000 for proteins A, G, or chimeric protein AG whose mean OD values are slightly above 2.0. However, the dilution factor 1:8000 for proteins for proteins A, G or chimeric protein AG was not considered in the study. **(B) Confirmation of appropriate dilutions of proteins.** Using the determined dilution factor for each protein, no significant differences were observed in the mean OD values of the animal sera (*p* > 0.05). The mean OD values of protein A and protein AG for pigs at dilution factors 1:128,000 and 1:16,000, respectively, were 2.06 and 2.09, while for dogs at the same dilution factors, the values were 2.03 and 2.11; similarly, the mean OD values of protein G and protein AG for cows at dilution factors 1:32,000 and 1:16,000, respectively, were 2.01 and 2.01, while for goats at the same dilution factors, the values were 1.97 and 2.05. Abbreviations: OD, optical density; ELISA, enzyme-linked immunosorbent assay.

First, ELISA was conducted using sera or plasma from pigs, cows, goats, or dogs to determine the dilution factor of proteins. To determine the dilution factor, different dilutions were performed. Dilutions 1:4000 to 1:128,000 were used to determine the dilution factor for three proteins using sera from pig, cow, goat, and dog plasma. The OD values of proteins A and AG were selected based on similar OD values for pig sera and dog plasma because protein A has been known to have a strong affinity for immunoglobulins in pigs and dogs [24]. Mean OD values were determined by plotting the graph, matching similar mean OD values against dilutions of the two proteins in pig sera and dog plasma. Following plotting and matching, the mean OD values for the dilution of proteins A and AG were 1:128,000 and 1:16,000, respectively (Fig 1A). In addition, no significant difference was observed between OD values 1:128,000 for the dilution of protein A and the 1:16,000 dilution of protein AG in pig sera and dog plasma (*p* > 0.05; Fig 1B).

The OD values of proteins G and AG were selected based on similar OD values for cow and goat sera because protein G has a strong affinity for immunoglobulins [18]. Mean OD values were calculated by plotting similar mean OD values against dilutions of proteins G and AG in cow and goat sera. Plotting and matching revealed similar mean OD values for proteins G and AG at dilutions of 1:32,000 and 1:16,000, respectively (Fig 1B). Moreover, there was no significant difference between the 1:32,000 dilution of protein G and the 1:16,000 dilution of protein AG in cow and goat sera (*p* > 0.05; Fig 1B).

As a result, for further experiments, the dilution factor of 1:128,000, 1:32,000, and 1:16,000 was used for proteins A, G, and AG, respectively.

Next, ELISA was carried out using the determined dilution factor of the proteins to detect the immunoglobulins of ten pinniped and five cetacean species. Sera from pinniped species revealed mean OD values between 0.76–2.47, 0.39–1.92, and 1.92–2.37 in proteins A, G, and AG, respectively (Table 1). Baikal seal, harbor seal, ringed seal, spotted seal, South American sea lion, Steller sea lion, and walrus serum samples revealed mean OD values ≥ 2.0 for protein A (Table 1). Protein G showed mean OD values less than 2.0 in pinniped sera (Table 1). Immunoglobulins of pinnipeds reacted more strongly to protein A than protein G. Sera from pinniped species showed mean OD values ≥ 2.0 in protein AG, except for the northern fur seal. Northern fur seal serum revealed the lowest mean OD values in proteins A and G. Also, pinniped immunoglobulins reacted strongly with chimeric protein AG.

**Table 1:** Marine mammal species used in this study and immunoglobulin-protein binding properties.

Marine mammal species	Scientific name	Number of samples tested	Mean OD values (± SD)		
			Protein A	Protein G	Protein AG
**Pinniped**					
Baikal seal	*Pusa sibirica*	4	2.33 (0.20)	1.91 (0.21)	2.01 (0.25)
California sea lion	*Zalophus californianus*	4	1.94 (0.03)	1.12 (0.08)	2.20 (0.05)
Harbor seal	*Phoca vitulina*	5	2.20 (0.19)	1.66 (0.24)	2.12 (0.08)
Northern fur seal	*Callorhinus ursinus*	5	0.76 (0.20)	0.39 (0.15)	1.92 (0.16)
Ringed seal	*Pusa hispida*	4	2.36 (0.09)	1.92 (0.13)	2.27 (0.03)
South American fur seal	*Arctocephalus australis*	5	1.65 (0.16)	0.92 (0.23)	2.28 (0.18)
South American sea lion	*Otaria flavescens*	4	2.47 (0.05)	1.08 (0.23)	2.37 (0.04)
Spotted seal	*Phoca largha*	5	2.42 (0.18)	1.91 (0.14)	2.26 (0.13)
Steller sea lion	*Eumetopias jubatus*	2	2.17 (0.15)	0.93 (0.20)	2.32 (0.04)
Walrus	*Odobenus rosmarus*	2	2.36 (0.03)	0.98 (0.11)	2.31 (0.10)
**Cetacean**					
Beluga whale	*Delphinapterus leucas*	5	1. 58 (0.16)	1.30 (0.14)	1.70 (0.08)
Bottlenose dolphin	*Tursipos truncatus*	5	1.80 (0.17)	0.09 (0.05)	1.40 (0.12)
Harbor porpoise	*Phocoena phocoena*	4	1.48 (0.17)	0.87 (0.17)	1.78 (0.16)
Killer whale	*Orcinus orca*	4	1.99 (0.17)	1.56 (0.23)	2.25 (0.11)
Pacific white-sided dolphin	*Lagenorhynchus obliquidens*	5	1.22 (0.17)	0.79 (0.24)	1.76 (0.12)

OD, optical density; SD, standard deviation; n, number.

Sera from cetacean species revealed mean OD values between 1.22–1.99, 0.09–1.56, and 1.40–2.25 in proteins A, G, and AG, respectively (Table 1). Sera from cetacean species revealed mean OD values ≥ 1.0 for proteins A and AG. Interestingly, the lowest mean OD value was observed in bottlenose dolphins for protein G (Table 1), indicating that immunoglobulins of bottlenose dolphins have a strong binding ability to protein A but lower ability to protein G. Killer whales had the highest mean OD value among the other cetaceans species for proteins A and AG. In contrast, beluga whales, harbor porpoises, killer whales, and Pacific white-sided dolphins had mean OD values ≥ 0.79 for protein G (Table 1). In this study, immunoglobulins of cetaceans reacted more strongly to protein A than protein G. Moreover, cetacean immunoglobulins reacted strongly with chimeric protein AG.

## Discussion

Previous studies have detected immunoglobulin binding abilities in wild animals using proteins A, G, and AG [23,24,26]. However, there are currently no comparative studies on the binding ability of immunoglobulins in marine mammals to proteins A, G, or AG. This study assessed the binding properties of pinniped and cetacean immunoglobulins to proteins A, G, and AG. The immunoglobulins of pinnipeds and cetaceans reacted more strongly to protein A than protein G and also reacted strongly to protein AG. This study will be useful for the development of serological assays for the serosurveillance of infectious diseases in pinnipeds and cetaceans.

The reactivity of protein A varies among immunoglobulin classes and sub-classes in animals [24]. In addition, protein A comprises more homologous immunoglobulin-binding domains than protein G [33,34], potentially indicating protein A has an increased affinity for immunoglobulins. In our study, protein A reacted more strongly with the immunoglobulins of pinnipeds and cetaceans and also chimeric protein AG reacted strongly to the immunoglobulins of pinnipeds and cetaceans. However, besides ten pinnipeds and five cetacean species used in this study, whether there are species having immunoglobulins bind strongly to protein G remain unclear. Further investigation is required.

The phylogenetic relatedness of the tested species may have played a role in the observed differences in the reactivity of immunoglobulins to proteins A and G [35]. Specifically, this study found that immunoglobulins from pinnipeds and cetaceans, which are classified into the orders Carnivora and Artiodactyla, respectively, reacted more strongly to protein A than protein G. However, there may be exceptions to this relationship, and protein A may also be effective in a wider range of animal species beyond just those in the same evolutionary order.

Zoonotic diseases can be transmitted from animals to humans and marine mammals are no exception. Although marine mammals do not come into direct contact with humans as often as terrestrial animals, a number of zoonotic diseases can be transmitted from marine mammals to humans. In this study, the immunoglobulins of pinniped and cetacean species showed stronger reactivity with protein A than with protein G and also reacted strongly with protein AG. These proteins can be used to develop assays further to detect antibodies against infectious agents in pinnipeds and cetaceans. Moreover, this ELISA method could be used to isolate immunoglobulins to see what isotypes are detected.

## Conclusion

To the best of our knowledge, this is the first comparative study to detect immunoglobulins in pinnipeds and cetaceans using proteins A, G, and the chimeric protein AG. This study provides a baseline for developing and optimizing serological assays using these bacterial proteins to monitor infections in marine mammals, which will contribute to the future risk of EIDs epidemics in humans from marine mammals.

## Supporting information

S1 Table OD values of the individual experiment. (DOCX)

S2 Table OD values of the individual experiment. (DOCX)

S3 Table General information on the pinniped species used in the study. (DOCX)

S4 Table General information on the cetacean species used in the study. (DOCX)
