# Supplementary material for: Assessing of the use of proteins A, G, and chimeric protein AG to detect marine mammal immunoglobulins
Source: PLoS One. 2023 Sep 21;18(9):e0291743. doi: 10.1371/journal.pone.0291743 (PMC10513184; doi:10.1371/journal.pone.0291743)
Supplement: S1 Table — (DOCX) [file pone.0291743.s001.docx]

# Supporting Information

| **S1 Table. OD values of the individual experiment** | | | | | | | | | | | |
| --- | --- | --- | --- | --- | --- | --- | --- | --- | --- | --- | --- |
| **Animal** | **Protein dilution** | **Protein A mean OD (± SD)** | | | |  | **Protein AG mean OD (± SD)** | | | | |
|  |  | **Experiment 1** | **Experiment 2** | **Experiment 3** | **Mean** |  | **Experiment 1** | **Experiment 2** | **Experiment 3** | | **Mean** |
| **Pig**  **(n = 4)** | 1:4000 | 2.44 (0.20) | 2.27 (0.19) | 2.45 (0.14) | 2.39 (0.10) |  | 2.14 (0.13) | 2.30 (0.01) | 2.38 (0.03) | 2.27 (0.12) | |
|  | 1:8000 | 2.48 (0.18) | 2.24 (0.18) | 2.45 (0.11) | 2.39 (0.13) |  | 2.15 (0.06) | 2.24 (0.01) | 2.31 (0.04) | 2.23 (0.08) | |
|  | 1:16,000 | 2.32 (0.30) | 2.38 (0.17) | 2.38 (0.12) | 2.36 (0.04) |  | 2.07 (0.03) | 1.98 (0.03) | 2.21 (0.03) | 2.09 (0.12) | |
|  | 1:32,000 | 2.19 (0.40) | 2.32 (0.20) | 2.34 (0.12) | 2.28 (0.08) |  | 1.78 (0.07) | 1.84 (0.03) | 1.64 (0.45) | 1.76 (0.10) | |
|  | 1:64,000 | 2.30 (0.14) | 2.25 (0.22) | 2.34 (0.13) | 2.29 (0.04) |  | 1.36 (0.02) | 1.37 (0.04) | 1.26 (0.38) | 1.33 (0.06) | |
|  | 1:128,000 | 2.13 (0.09) | 2.05 (0.16) | 2.00 (0.19) | 2.06 (0.07) |  | 0.88 (0.07) | 0.85 (0.04) | 0.92 (0.05) | 0.88 (0.04) | |
| **Dog**  **(n = 4)** | 1:4000 | 2.45 (0.09) | 2.40 (0.16) | 2.50 (0.08) | 2.45 (0.05) | | 2.27 (0.01) | 2.31 (0.01) | 2.30 (0.05) | 2.29 (0.90) | |
|  | 1:8000 | 2.38 (0.15) | 2.30 (0.24) | 2.51 (0.06) | 2.40 (0.11) | | 2.21 (0.02) | 2.24 (0.03) | 2.25 (0.02) | 2.23 (0.02) | |
|  | 1:16,000 | 2.46 (0.11) | 2.38 (0.16) | 2.47 (0.02) | 2.44 (0.05) | | 2.10 (0.03) | 2.11 (0.03) | 2.11 (0.03) | 2.11 (0.00) | |
|  | 1:32,000 | 2.48 (0.15) | 2.31 (0.17) | 2.37 (0.05) | 2.39 (0.09) | | 1.86 (0.04) | 1.79 (0.07) | 1.82 (0.08) | 1.82 (0.04) | |
|  | 1:64,000 | 2.36 (0.25) | 2.27 (0.11) | 2.38 (0.05) | 2.34 (0.06) | | 1.43 (0.07) | 1.31 (0.04) | 1.40 (0.11) | 1.38 (0.06) | |
|  | 1:128,000 | 2.09 (0.29) | 1.96 (0.08) | 2.05 (0.22) | 2.03 (0.07) | | 0.89 (0.08) | 0.82 (0.03) | 0.89 (0.08) | 0.87 (0.04) | |

OD, optical density; SD, standard deviation; n, number
