# Supplementary material for: Assessing of the use of proteins A, G, and chimeric protein AG to detect marine mammal immunoglobulins
Source: PLoS One. 2023 Sep 21;18(9):e0291743. doi: 10.1371/journal.pone.0291743 (PMC10513184; doi:10.1371/journal.pone.0291743)
Supplement: S2 Table — (DOCX) [file pone.0291743.s002.docx]

| **S2 Table. OD values of the individual experiment.** | | | | | | | | | | | |
| --- | --- | --- | --- | --- | --- | --- | --- | --- | --- | --- | --- |
| **Animal** | **Protein dilution** | **Protein G mean OD (± SD)** | | | |  | **Protein AG mean OD (± SD)** | | | | |
|  |  | **Experiment 1** | **Experiment 2** | **Experiment 3** | **Mean** |  | **Experiment 1** | **Experiment 2** | **Experiment 3** | **Mean** |  |
| **Cow**  **(n = 4)** | 1:4000 | 2.14 (0.04) | 2.20 (0.03) | 2.21 (0.03) | 2.18 (0.04) |  | 2.20 (0.26) | 2.31 (0.03) | 2.11 (0.03) | 2.21 (0.10) |  |
|  | 1:8000 | 2.18 (0.04) | 2.18 (0.02) | 2.22 (0.02) | 2.19 (0.02) | | 2.09 (0.16) | 2.23 (0.01) | 2.10 (0.02) | 2.14 (0.08) |  |
|  | 1:16,000 | 2.14 (0.01) | 2.04 (0.03) | 2.18 (0.05) | 2.12 (0.07) | | 1.89 (0.13) | 2.13 (0.02) | 2.00 (0.02) | 2.01 (0.12 |  |
|  | 1:32,000 | 1.99 (0.09) | 1.96 (0.01) | 2.09 (0.02) | 2.01 (0.07) | | 1.73 (0.12) | 1.91 (0.05) | 1.79 (0.03) | 1.81 (0.10) |  |
|  | 1:64,000 | 1.81 (0.03) | 1.82 (0.02) | 1.87 (0.03) | 1.83 (0.03) | | 1.47 (0.06) | 1.54 (0.04) | 1.42 (0.08) | 1.48 (0.06) |  |
|  | 1:128,000 | 1.34 (0.02) | 1.31 (0.01) | 1.35 (0.02) | 1.33 (0.02) | | 0.98 (0.04) | 1.07 (0.03) | 0.97 (0.08) | 1.01 (0.05) |  |
| **Goat**  **(n = 4)** | 1:4000 | 2.41 (0.23) | 2.45 (0.06) | 2.55 (0.05) | 2.47 (0.07) |  | 2.17 (0.03) | 2.23 (0.02) | 2.21 (0.04) | 2.20 (0.03) |  |
|  | 1:8000 | 2.16 (0.14) | 2.42 (0.15) | 2.49 (0.06) | 2.36 (0.17) | | 2.13 (0.07) | 2.18 (0.03) | 2.21 (0.03) | 2.17 (0.04) |  |
|  | 1:16,000 | 2.17 (0.26) | 2.35 (0.07) | 2.29 (0.10) | 2.27 (0.09) | | 2.05 (0.05) | 2.01 (0.02) | 2.08 (0.02) | 2.05 (0.04) |  |
|  | 1:32,000 | 1.88 (0.31) | 2.04 (0.17) | 1.98 (0.07) | 1.97 (0.09) | | 2.02 (0.02) | 1.71 (0.17) | 1.85 (0.03) | 1.86 (0.15) |  |
|  | 1:64,000 | 1.59 (0.11) | 1.66 (0.17) | 1.28 (0.40) | 1.51 (0.20) | | 1.48 (0.05) | 1.34 (0.08) | 1.47 (0.09) | 1.43 (0.08) |  |
|  | 1:128,000 | 1.23 (0.13) | 1.14 (0.12) | 0.97 (0.11) | 1.12 (0.13) | | 1.40 (0.01) | 0.88 (0.05) | 1.00 (0.09) | 1.09 (0.27) |  |

OD, optical density; SD, standard deviation; n, number
