# Supplementary material for: Assessing of the use of proteins A, G, and chimeric protein AG to detect marine mammal immunoglobulins
Source: PLoS One. 2023 Sep 21;18(9):e0291743. doi: 10.1371/journal.pone.0291743 (PMC10513184; doi:10.1371/journal.pone.0291743)
Supplement: S3 Table — (DOCX) [file pone.0291743.s003.docx]

| **S3 Table.** **General information on the pinniped species used in this study** | | | | | |  |
| --- | --- | --- | --- | --- | --- | --- |
| **Marine mammal species** | **ID** | **Age (years) ^*1^** | **Date of blood collection** | **Clinical condition ^*2^** | **Frozen period (years) ^*3^** |  |
|  |  |  |  |  |  |  |
| Baikal seal | 1 | 19 | 2023/01 | unknown | within 1 |  |
|  | 2 | 19 | 2019/05 |  | > 3 |  |
|  | 3 | 19 | 2022/12 |  | > 1 |  |
|  | 4 | 39 | 2017/09 |  | > 5 |  |
| California sea lion | 1 | 12 | 2022/12 | unknown | > 1 |  |
|  | 2 | 7 | 2022/11 |  |  |  |
|  | 3 | 11 | 2022/11 |  |  |  |
|  | 4 | 12 | 2022/11 |  |  |  |
| Harbor seal | 1 | – | 2022/12 | unknown | > 1 |  |
|  | 2 | – | 2022/12 |  |  |  |
|  | 3 | – | 2022/11 |  |  |  |
|  | 4 | – | 2022/11 |  |  |  |
|  | 5 | – | 2022/11 |  |  |  |
| Northern fur seal | 1 | 13 | 2022/05 | unknown | > 1 |  |
|  | 2 | 12 | 2022/05 |  |  |  |
|  | 3 | 6 | 2022/05 |  |  |  |
|  | 4 | 4 | 2022/05 |  |  |  |
|  | 5 | 3 | 2022/05 |  |  |  |
| Ringed seal | 1 | 15 | 2022/10 | unknown | > 1 |  |
|  | 2 | 14 | 2022/10 |  |  |  |
|  | 3 | 17 | 2022/11 |  |  |  |
|  | 4 | 1 | 2022/12 |  |  |  |

**^*1^**Age, age at the time of blood collection (– means not provided); **^2^** Clinical condition, condition of marine mammals at the time of blood collection; **^*3^** frozen period (years), the time the serum samples were stored after blood collection.

| **S3 Table. Continued** | | | | | |  |
| --- | --- | --- | --- | --- | --- | --- |
| **Marine mammal species** | **ID** | **Age (years) ^*1^** | **Date of blood collection** | **Clinical condition ^*2^** | **Frozen period (years) ^*3^** |  |
|  |  |  |  |  |  |  |
| South American fur seal | 1 | 14 | 2022/11 | good health | > 1 |  |
|  | 2 | 12 | 2023/01 | good health |  |  |
|  | 3 | 12 | 2022/11 | good health |  |  |
|  | 4 | 9 | 2022/10 | good health |  |  |
|  | 5 | 13 | 2022/10 | mild inflammatory tendency |  |  |
| South American sea lion | 1 | – | 2022/08 | unknown | > 1 |  |
|  | 2 | – | 2022/08 |  |  |  |
|  | 3 | – | 2022/08 |  |  |  |
|  | 4 | – | 2022/08 |  |  |  |
| Spotted seal | 1 | 13 | 2021/10 | unknown | > 1 |  |
|  | 2 | 12 | 2021/10 | unknown |  |  |
|  | 3 | 15 | 2022/10 | good health |  |  |
|  | 4 | 13 | 2022/10 | good health |  |  |
|  | 5 | 23 | 2022/10 | good health |  |  |
| Steller sea lion | 1 | – | 2022/11 | unknown | > 1 |  |
|  | 2 | – | 2022/09 | unknown | > 1 |  |
| Walrus | 1 | – | 2022/08 | unknown | > 1 |  |
|  | 2 | 25 | 2022/01 | unknown | > 1 |  |

**^*1^**Age, age at the time of blood collection (– means not provided); **^*2^** Clinical condition, condition of marine mammals at the time of blood collection; **^*3^** frozen period (years), the time the serum samples were stored after blood collection.
