# Supplementary material for: Assessing of the use of proteins A, G, and chimeric protein AG to detect marine mammal immunoglobulins
Source: PLoS One. 2023 Sep 21;18(9):e0291743. doi: 10.1371/journal.pone.0291743 (PMC10513184; doi:10.1371/journal.pone.0291743)
Supplement: S4 Table — (DOCX) [file pone.0291743.s004.docx]

| **S4 Table.** **General information on the cetacean species used in this study** | | | | | | |  |
| --- | --- | --- | --- | --- | --- | --- | --- |
| **Marine mammal species** | **ID** | **Age (years) ^*1^** | | **Date of blood collection** | **Clinical condition^*2^** | **Frozen period (years) ^*3^** |  |
|  |  |  |  |  |  |  |  |
| Beluga whale | 1 | 23 | 2022/01 | | unknown | > 1 |  |
|  | 2 | 22 | 2021/12 | |  |  |  |
|  | 3 | 25 | 2022/01 | |  |  |  |
|  | 4 | 15 | 2022/01 | |  |  |  |
|  | 5 | 12 | 2022/01 | |  |  |  |
| Bottlenose dolphin | 1 | 23 | 2022/07 | | unknown | > 1 |  |
|  | 2 | 20 | 2022/08 | |  |  |  |
|  | 3 | 19 | 2022/07 | |  |  |  |
|  | 4 | 23 | 2022/08 | |  |  |  |
|  | 5 | 15 | 2022/07 | |  |  |  |
| Harbor porpoise | 1 | – | 2022/11 | | unknown | > 1 |  |
|  | 2 | – | 2022/08 | |  |  |  |
|  | 3 | – | 2022/07 | |  |  |  |
|  | 4 | – | 2022/11 | |  |  |  |
| Killer whale | 1 | 18 | 2022/12 | | unknown | > 1 |  |
|  | 2 | 12 | 2022/12 | |  | > 1 |  |
|  | 3 | 12 | 2022/01 | |  | > 1 |  |
|  | 4 | 14 | 2021/12 | |  | > 2 |  |
| Pacific white-sided dolphin | 1 | 17 | 2022/07 | | unknown | > 1 |  |
|  | 2 | – | 2022/07 | |  | > 1 |  |
|  | 3 | 16 | 2021/04 | |  | > 2 |  |
|  | 4 | 14 | 2021/12 | |  | > 2 |  |
|  | 5 | 13 | 2021/12 | |  | > 2 |  |

**^*1^**Age, age at the time of blood collection (– means not provided); **^*2^** Clinical condition, condition of marine mammals at the time of blood collection; **^*3^** frozen period (years), the time the serum samples were stored after blood collection.
